# High-Resolution Mass Spectrometry-Based Chemical Fingerprinting of Baijiu, a Traditional Chinese Liquor

**DOI:** 10.1021/acsomega.3c08993

**Published:** 2024-02-13

**Authors:** Yanning Dou, Marko Mäkinen, Janne Jänis

**Affiliations:** Department of Chemistry, University of Eastern Finland, P.O. Box 111, Joensuu FI-80101, Finland

## Abstract

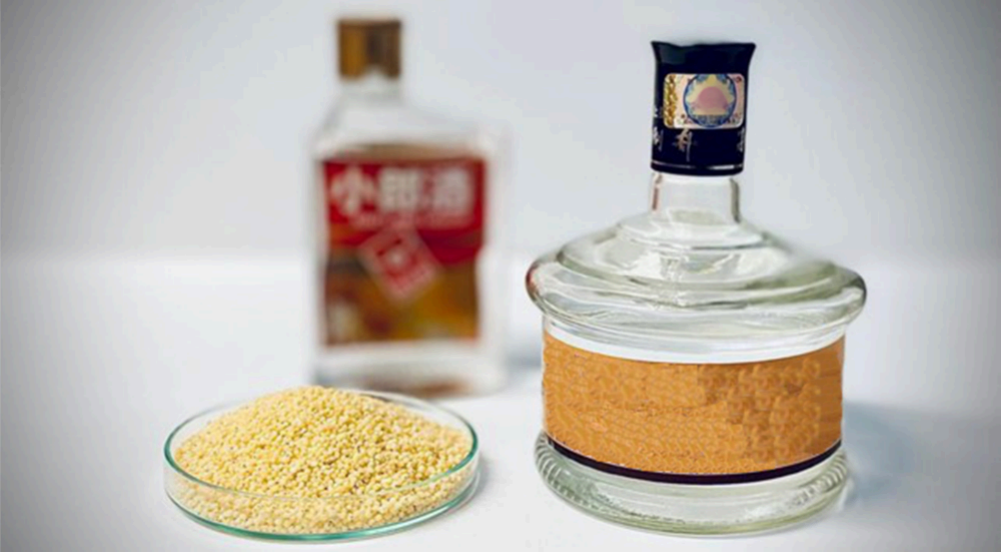

Fourier transform ion cyclotron resonance (FT-ICR) mass spectrometry, coupled with electrospray ionization (ESI) or atmospheric-pressure photoionization (APPI), was employed for chemical fingerprinting of baijiu, a traditional Chinese liquor. Baijiu is the most consumed distilled alcoholic beverage globally, with over 10 billion liters sold annually. It is a white (transparent) spirit that exhibits similarities to dark spirits such as whisky or rum in terms of aroma and mouthfeel. In this study, direct-infusion FT-ICR mass spectrometry was used to analyze 10 commercially available baijiu liquors, enabling the examination of both volatile and nonvolatile constituents without the need for tedious sample extractions or compound derivatizations. The chemical fingerprints obtained by FT-ICR MS revealed substantial compositional diversity among different baijiu liquors, reflecting variations in the raw materials and production methods. The main compounds identified included a variety of acids, esters, aldehydes, lactones, terpenes, and phenolic compounds. The use of ESI and APPI provided complementary compositional information; while ESI demonstrated greater selectivity toward polar, aliphatic sample constituents, APPI also ionized semipolar and nonpolar (aromatic) ones.

## 1 Introduction

Baijiu, a national drink of China, is a transparent or yellowish distilled spirit with more than 1000 years of history.^1,2^ As a world-renowned alcoholic product, baijiu is the most consumed distilled spirit, with a net market value reaching $100 billion in 2016.^1^ Thus, it is also one of the major contributors to the Chinese food industry. Traditional raw materials for baijiu making are smashed sorghum and wheat, which are fermented by using *jiuqu* (also known as *qu*), a starter of fermentation; then, the fermentation mixture is distilled to produce the final liquor.^3,4^ The raw materials for Chinese liquor making can also be other grains like rice, barley, or corn.^5^ Baijiu has various flavors according to different raw materials used as well as the aging time.^6,7^ Most baijiu liquors are aged for about 1 year in porcelain jars, while premium baijiu brands are aged for more than 3 years.^7^ Most commonly, baijiu liquors are divided into four main classes (or flavor types) based on their aroma profiles: light aroma (*qingxiang*), rice aroma (*mixiang*), strong aroma (*nongxiang*), and sauce aroma (*jiangxiang*). In addition, there are several other niche classes and regional varieties. Although baijiu is a white (transparent) distilled spirit, it resembles common dark alcoholic beverages, such as whisky or rum, in terms of its aroma and mouthfeel. The ethanol content of baijiu typically ranges from 30 to 60% alcohol by volume (ABV).

Baijiu contains a large number of compounds, resulting from the raw materials and the fermentation and distillation processes applied.^5,8−14^ Organic acids, esters, aldehydes, ketones, lactones, higher alcohols, phenolic compounds, nitrogen heterocycles, sulfur-containing compounds, and terpenes are among the most abundant ones.^5,8−14^ For instance, Fan and Qian identified ethyl esters of butanoic, pentanoic, hexanoic, and octanoic acids as well as butyl hexanoate, ethyl 3-methylbutanoate, hexanoic acid, and diethoxy-3-methylbutane as the main flavor substances in strong aroma baijiu.^15^ These compounds contributed mainly to the fruity-like aroma with the exception that hexanoic acid imparts a sweaty note.^15^ In the same study, several alkylpyrazines were also identified.^15^ Another work reported ethyl butanoate, ethyl pentanoate, and ethyl hexanoate as the most abundant compounds in some samples, suggesting that (straight-chain) esters are the main aroma contributors in baijiu.^16^ Ding et al. analyzed the vapors of the Luzhou-flavor baijiu fermentation cellars directly by headspace solid-phase microextraction (HS-SPME) connected to gas chromatography–mass spectrometry (GC–MS). They identified 59 volatile organics, with the most abundant being phenylethyl alcohol, ethyl lactate, ethyl hexanoate, ethyl hexadecanoate, ethyl linoleate, and ethyl elaidate as well as short-chain fatty acids.^17^ In a recent study, Sun et al. reported ethyl pentanoate, 3-methyl-1-butanol, methional, ethyl 3-phenylpropanoate, and 2-phenylethanol as the compounds with the highest flavor dilution (FD) factors in the Meilanchun baijiu, while the compounds of the highest concentrations were ethyl acetate, ethyl lactate, ethyl propanoate, ethyl 3-phenylpropanoate, butyl hexanoate, acetic acid, decanoic acid, and lactic acid.^18^ The FD factor is a measure of the relative importance of each odorant to the perceived flavor of the product and is assessed by aroma extract dilution analysis (AEDA).

Moreover, baijiu has some potential bioactive compounds such as peptides and free amino acids.^19,20^ In addition, many volatile sulfur-containing compounds (VSCs) are present in baijiu, such as dimethyl disulfide/trisulfide, 2-furfurylthiol, and 2-methyl-3-furanthiol.^12,21^ Furthermore, different pyrazines are among the important aroma compounds in baijiu. Fan et al. identified a total of 27 pyrazines in 12 commercial baijiu liquors, with different alkyl- and acetylpyrazines being the most abundant.^22^ While there are several different compound classes that contribute to the complex flavor profiles of baijiu liquors, different studies suggest that esters are the most important flavor substances. A recent study lists over 500 esters identified in baijiu samples and further discusses their importance to the distinct flavor types.^23^ Similarly, nonvolatile organic acids strongly contribute to the baijiu flavor profiles and could also be used to differentiate between different types of products.^24^

The main aroma compounds in baijiu have been identified by a gas chromatography–olfactometry (GC–O) method in several studies.^15,16,25−27^ In addition, GC–MS has been commonly used for quantitative compound screening.^22,28,29^ GC–MS and GC–O have also been applied together as complementary techniques.^15,16,25^ For GC–MS analysis, either liquid–liquid extraction (LLE) or HS-SPME is commonly applied to avoid nonvolatile compounds and water entering into the GC injector and the column. Another approach is the use of electronic noses or tongues; they have been used to classify Chinese liquors of the same aroma style^30^ and for quality assessments.^31^ A colorimetric artificial nose was used to identify baijiu liquors based on their geographic origins^32^ or brands.^33^ However, these studies are highly targeted, and there are unavoidable drawbacks with these techniques such as complicated sample pretreatments (compound extractions and/or derivatizations), time-consuming operations, and inaccurate data resulting from low-resolution conventional instruments.

In the present study, direct-infusion (DI) Fourier transform ion cyclotron resonance (FT-ICR) mass spectrometry was applied to Chinese baijiu by using both negative-ion electrospray ionization (ESI) and positive-ion atmospheric-pressure photoionization (APPI). The FT-ICR MS technique is a powerful analytical tool for complex mixture analysis, especially useful for nontargeted metabolomic studies.^12^ In the context of alcoholic beverages, DI FT-ICR MS has been previously applied to the compositional analyses of whisky,^34−36^ wine,^37,38^ and more recently gin.^39^ The main advantage of the FT-ICR technique is its unparalleled mass resolving power, allowing for a confident assignment of tens of thousands of compounds in a single run. Moreover, a DI approach enables analysis of dilute aqueous solutions of organic samples without any sample pretreatments or compound derivatizations—perfect for foodomic applications. Therefore, it is very well suited for nontargeted chemical fingerprinting of complex organic mixtures such as alcoholic beverages. Very recently, ESI FT-ICR MS was used for the identification of trace components in six sauce flavor baijiu samples.^12^ Our main goal in this study was to apply FT-ICR MS for chemical profiling of various commercial baijiu samples while coupling the technique with two complementary ionization techniques (ESI and APPI) to cover a wider range of polar and nonpolar (aliphatic and aromatic) compounds.

## 2 Materials and Methods

### 2.1 Chemicals and Sample Preparation

Ten commercially available baijiu liquors (B1–B10; see Table S1 for details) were purchased from a local supermarket in China. For the DI mass spectrometry analysis, the samples were prepared as follows: for the negative-ion ESI measurements, 100 μL of each baijiu liquor was diluted with 900 μL of HPLC-grade methanol, and 5 μL of 1 M ammonium hydroxide was added; for the positive-ion APPI measurements, 100 μL of each liquor was diluted with 900 μL of a methanol/toluene mixture (9:1, v/v). The same solvent mixtures served as the negative control samples (solvent blanks). All solvents and reagents were of HPLC grade. To avoid any carryover between the samples, the sample transfer capillary and the sample syringe were washed three times with methanol/water/acetic acid (1:1 v/v + 1%) followed by methanol/water (1:1, v/v), before the new sample was introduced.

### 2.2 Mass Spectrometry

All mass spectrometric experiments were performed with a 12 T Bruker solariX XR FT-ICR instrument (Bruker Daltonics GmbH, Bremen, Germany) equipped with a dynamically harmonized ICR cell (ParaCell) and an Apollo-II ESI/APPI-II ion source. The diluted baijiu samples were directly infused into the ion source at a flow rate of 2 μL/min for ESI or 5 μL/min for APPI. The drying gas (nitrogen) flow rate was 4.0 L/min, and the temperature was 220 °C. For each spectrum, a sum of 300 time-domain transients was recorded prior to the fast Fourier transform and magnitude calculation. A full-sine apodization was applied, and the transients were zero-filled once to provide the final 16 MWord magnitude-mode data at an *m*/*z* range of 90–1000 (the resolving power was ∼780,000 FWHM at *m*/*z* 400). Bruker ftmsControl 2.1 software was used for data acquisition and instrument control.

The FT-ICR MS data were further processed and analyzed using Bruker DataAnalysis 5.0 software. For the peak assignments, the signal-to-noise (S/N) ratio was ≥5 and the relative intensity threshold was 0.01%. The internal mass recalibration was performed with custom-made calibration lists, resulting in an RMS mass error of <100 ppb. The elemental formulas for the peaks were obtained against the elemental space of ^12^C_0–100_^1^H_0–300_^16^O_0–30_^14^N_0–2_^32^S_0–__1_. For the SmartFormula peak assignment, the following constraints were applied: mass error, ≤1.0 ppm; maximum number of formulas, ≤50; double bond equivalent (DBE), ≤80; H/C ratio, ≤3; electron configuration, even (ESI), even/odd (APPI); and mSigma value ≤1000. The initial data sorting was performed using Microsoft Excel software (Microsoft Corporation, Redmond, WA, United States). A further data visualization was made with the Ceres Viewer 1.82 software (University of Rostock, Germany).

A CompoundCrawler database search engine was used to facilitate putative compound identifications. In instances in which multiple candidates were obtained for a given molecular formula, a thorough examination of compounds reported in earlier studies was conducted to propose the most likely structure(s). The majority of compound identifications fell under “confidence level 3 or 4” (i.e., unique molecular formula or tentative structure), as previously recommended by Schrimpe-Rutledge et al.^40^ Specifically, in the context of esters, numerous potential candidates exist for a given carbon number. Therefore, all previous identifications based on GC–MS were scrutinized to propose the most likely constitutional isomer in each case.

## 3 Results and Discussion

### 3.1 Negative-Ion ESI FT-ICR MS Analyses

**Figure 1:**
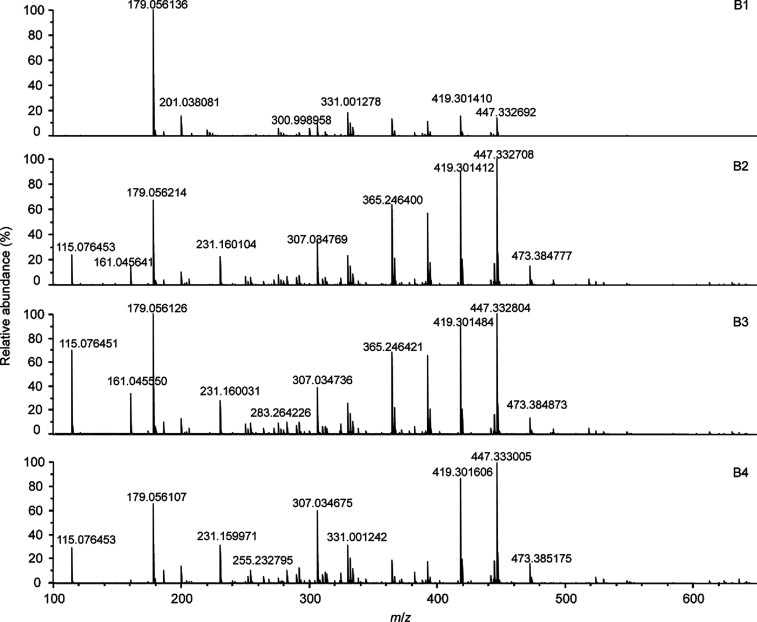
Negative-ion ESI-FT-ICR mass spectra of selected baijiu samples B1–B4.

Figure 1 shows the (−)ESI FT-ICR mass spectra of the four selected baijiu samples (for the spectra of the other samples, see Figure S1). All of the spectra had notable similarities but also distinct differences. At an S/N ratio of ≥5, approximately 500–700 spectral features (unique molecular formulas) could be assigned for baijiu samples B1–B10 with negative-ion ESI.

**Figure 2:**
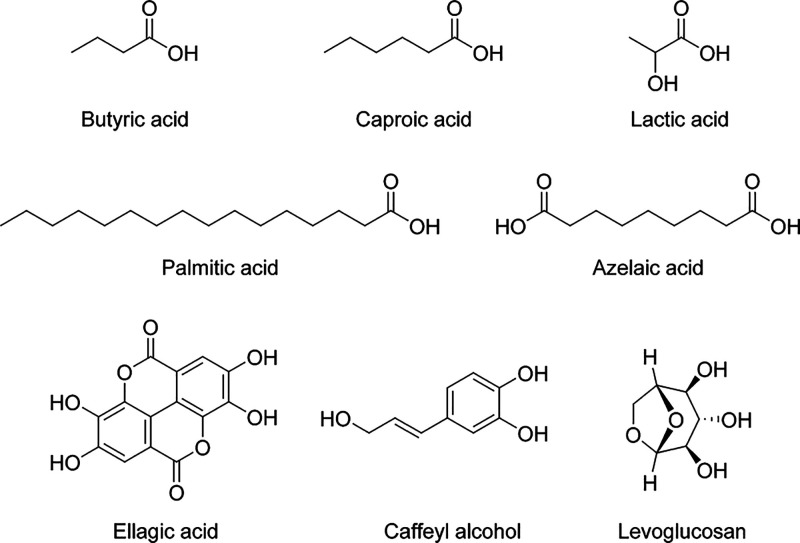
Proposed structures for some selected compounds detected in the baijiu samples with (−) ESI.

Table 1 gives a summary of the identified compounds in all studied baijiu samples using negative-ion ESI. The identified compounds mainly included different organic (fatty) acids and carbohydrates as well as their derivatives. In addition, some phenolic compounds were observed as well. The structures of some selected compounds are presented in Figure 2.

**Table 1:**
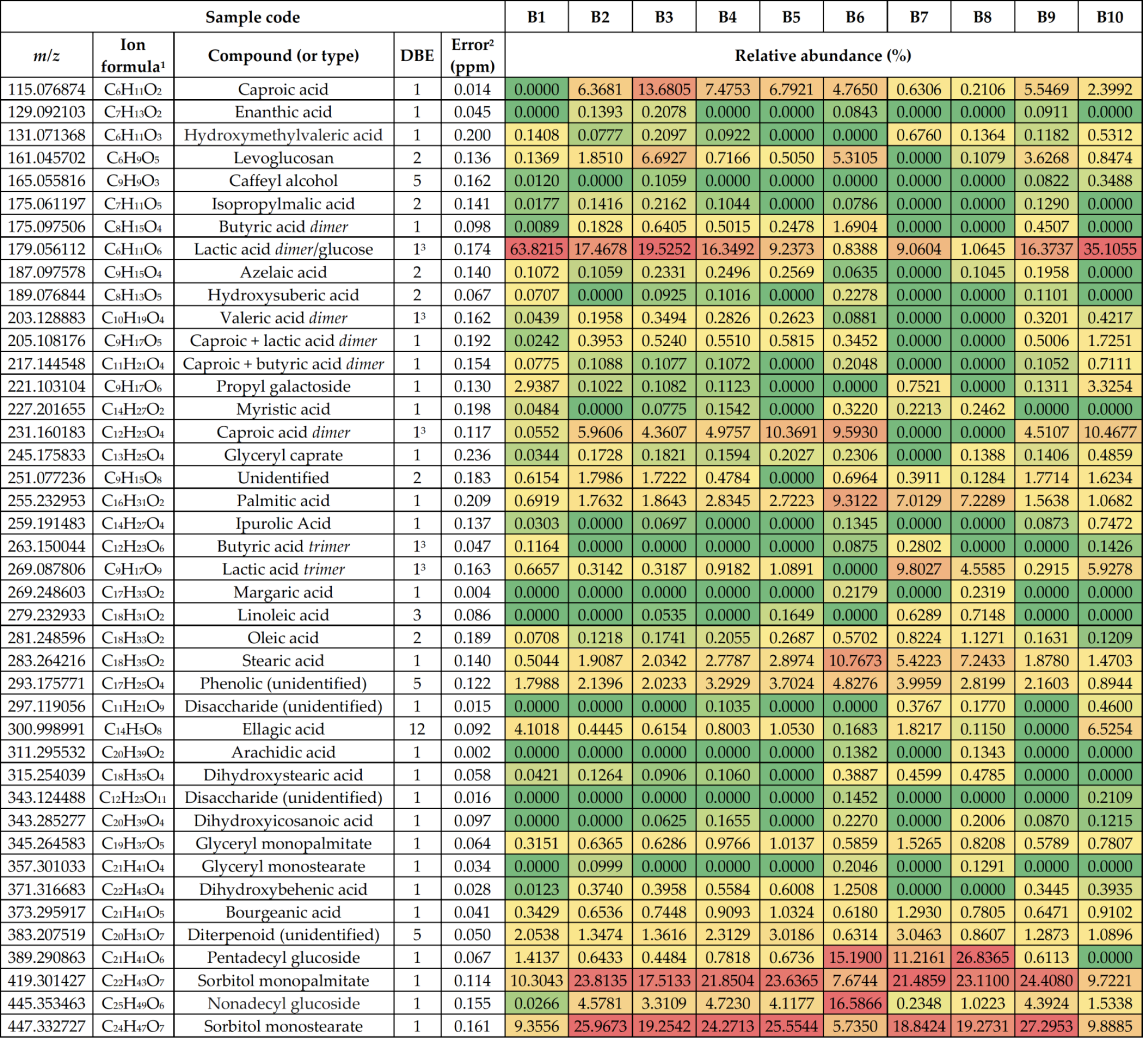
Summary of the Most Abundant Compounds Tentatively Identified in the Baijiu Samples by (−)ESI FT-ICR MS

1 For the deprotonated molecule.

2 RMS error for the 10 baijiu samples.

3 For the monomer.

Out of all acids detected, the highest abundance was observed for lactic acid (2-hydroxypropanoic acid, C_3_H_6_O_3_; Figure 2), which was observed as a noncovalent dimer at *m*/*z* 179.056112. A tendency for strong dimerization of lactic acid under the solution conditions used was further confirmed by separate DI measurements with a pure reference standard (Figure S2). Lactic acid has been reported as the most abundant among the nonvolatile organic acids (NVOAs) in baijiu^24^ with up to an ∼2 g/L concentration in some product varieties. The lactic acid monomer was observed at *m*/*z* 89 (in negative-ion ESI), which is below the mass (*m*/*z*) range with the instrument parameters used for baijiu samples. Similarly, the smallest (volatile) organic acids (<100 Da) were not detected either. A tendency of short-chain NVOA dimerization was also observed with the other acids as well. The second most abundant fatty acid was caproic acid (hexanoic acid), observed as a monomer and a dimer. Other prevalent NVOAs in baijiu are dihydroxypropionic acid, hydroxymethylcaproic acid, benzoic acid, azelaic acid (nonanedioic acid), lauric acid, and long-chain fatty acid,^24^ and they were also detected in our study. It is worth mentioning that hexoses (e.g., glucose, C_6_H_12_O_6_) have the same exact mass as the lactic acid dimer and therefore cannot be readily differentiated. Trace amounts of sugars can be present in distilled spirits, especially if natured in wooden casks or sweetened after distillation. Some interesting surfactant molecules (i.e., two alkyl glucosides as well as four fatty acid esters of glycerol and sorbitol) were also identified in all baijiu samples. The latter compounds are typically used in the food industry as emulsifiers or stabilizers, for example, in different dairy products. The origin of these compounds in baijiu samples is unknown, however.

### 3.2 Positive-Ion APPI FT-ICR MS Analyses

**Figure 3:**
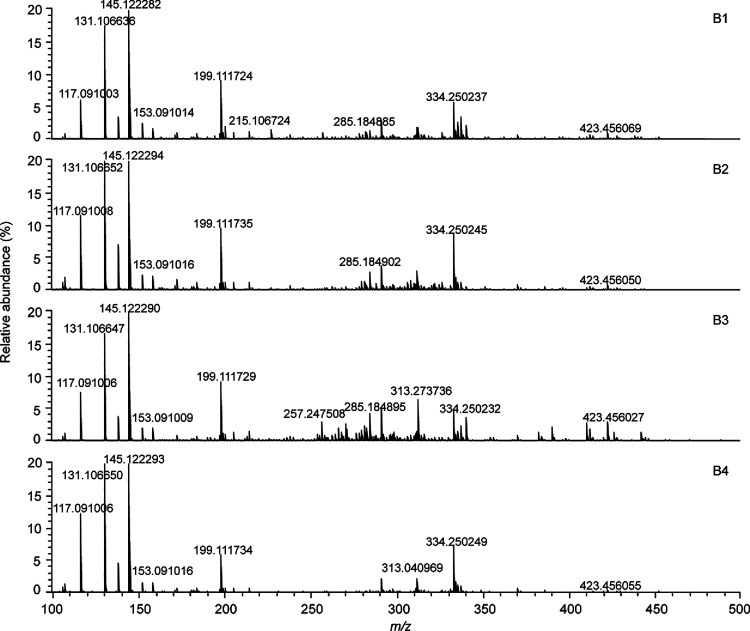
Positive-ion APPI FT-ICR mass spectra of selected baijiu samples B1–B4.

**Figure 4:**
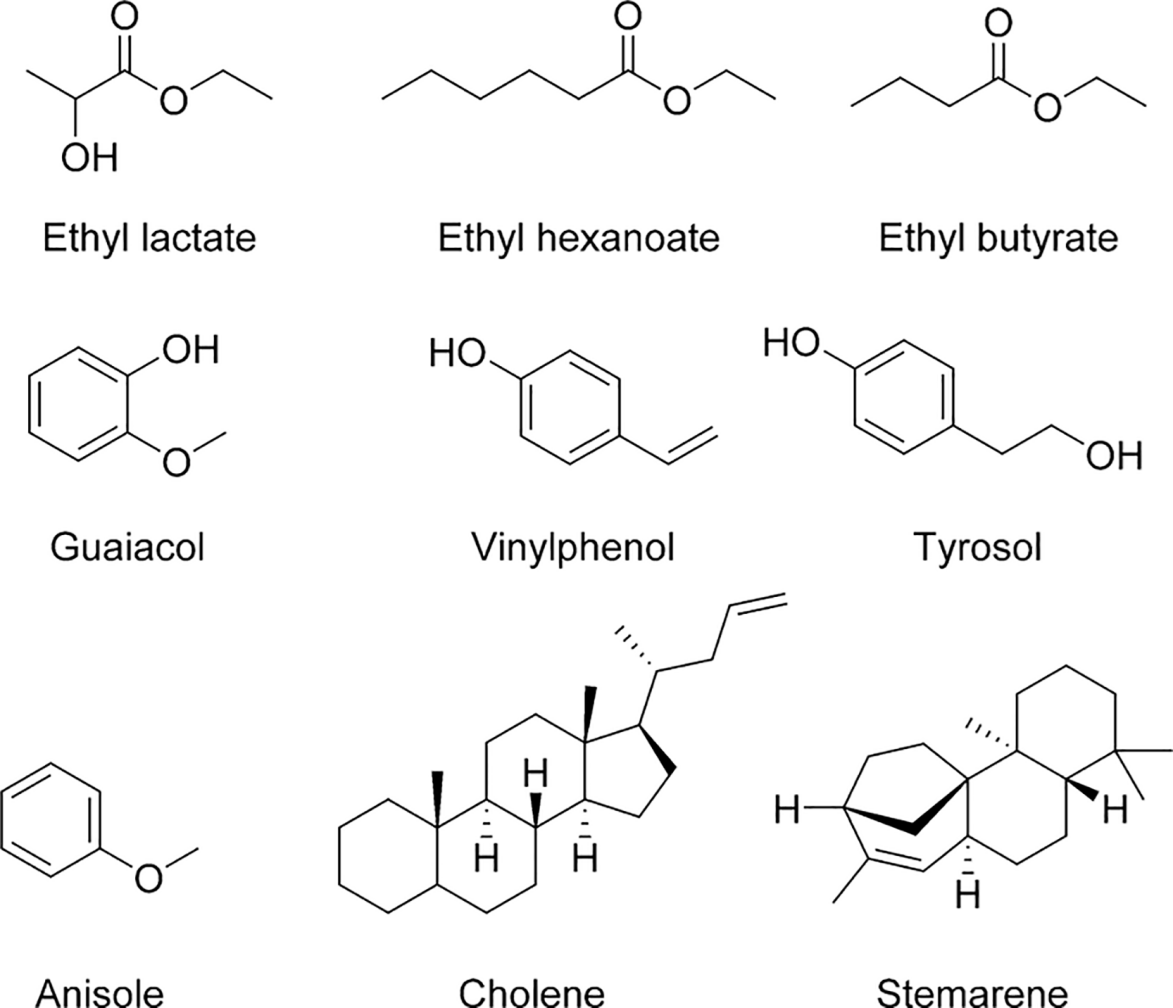
Proposed structures for some selected compounds detected in the baijiu samples with (+)APPI.

To effectively ionize semipolar and nonpolar constituents as well, additional mass spectral analyses were also performed with baijiu samples using positive-ion APPI, which preferentially targets aromatic and condensed, nonaromatic compounds. Figure 3 shows (+)APPI FT-ICR mass spectra obtained for the four selected baijiu samples (for the spectra of the other samples, see Figure S3). There were roughly 1000–1500 unique spectral features at an S/N ratio of ≥5, when considering even-electron ions only. The (+)APPI FT-ICR mass spectra indicated great similarities among all studied baijiu liquors. The compound observed at *m*/*z* 145.122282 was tentatively identified as ethyl hexanoate (ethyl caproate), which possessed the highest relative abundance in all studied samples. Ethyl hexanoate has been reported as the most frequently identified organic ester in different baijiu liquors.^41^ It is especially abundant in the strong aroma baijiu, for which it gives fruity notes (e.g., apple, pineapple, and banana). In addition, other alkyl esters were also observed, and they are considered the most important flavor compounds in baijiu. The other identified compounds include lactones, aldehydes, ethers, phenolic compounds, carbohydrates, alcohols, and some terpene hydrocarbons. The summary of the identified compounds can be found in Table 2 and the structures for some selected compounds in Figure 4. Since some compounds formed both radical cations and protonated molecules, Table S2 lists those where the former ion type was predominant. The average DBE values indicate that for protonated molecules, the average DBE value was 3.6, whereas for radical cations, it was 5.6. This suggests a preference for the formation of radical cations with (aromatic) compounds.

**Table 2:**
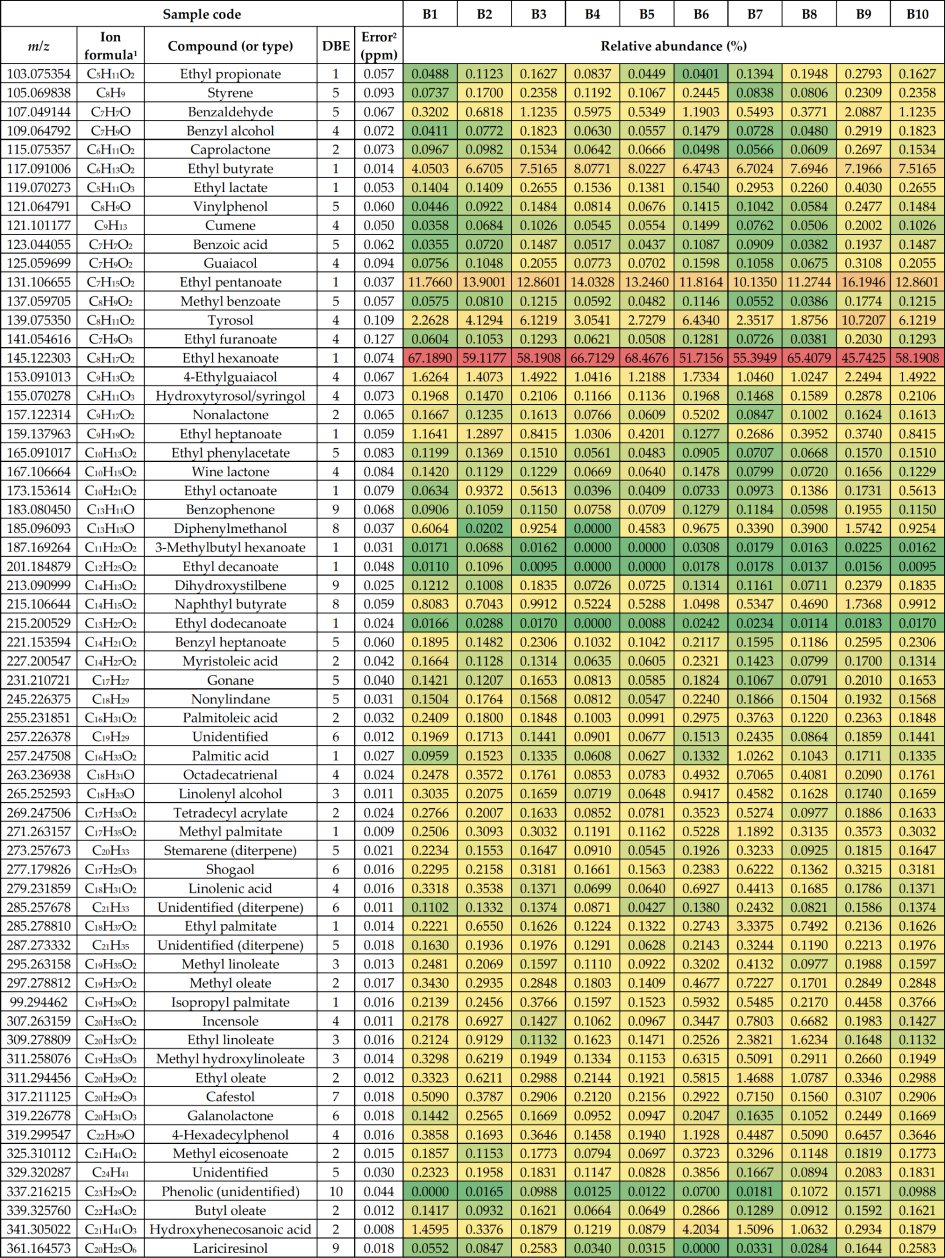
Summary of the Most Abundant Compounds Tentatively Identified in the Baijiu Samples by (+)APPI FT-ICR MS

1 For the protonated molecule.

2 RMS error for the 10 baijiu samples.

In contrast to ESI, terpene hydrocarbons were also detected with (+)APPI, some of which are difficult to identify solely based on the elemental formulas. A lot of phenolic compounds were also detected. For example, tyrosol (4-hydroxyphenylethanol) was abundantly present in all studied samples, which is a very prevalent phenolic compound in many foodstuff like vegetables and fruits. In addition, guaiacol, vinylphenol, ethylguaiacol, hydroxytyrosol, dodecylphenol, and shogaol were detected as well. Many of these compounds have not been previously detected, possibly due to the selective extraction methods used or the limitations of the analysis methods applied. Some may also be present in only trace amounts.

### 3.3 Overall Visualization of the Compounds Detected with ESI and APPI

A van Krevelen (VK) diagram is an effective visual means for the overall representation of the chemical composition of any complex organic mixture containing certain heteroatomic compounds. Typically, a VK diagram is a plot of the atomic H/C ratio against the O/C ratio for every compound in the mixture (for the oxygen-containing species), providing a way to classify compounds based on their residence in the two-dimensional diagram. In addition, the compound’s relative abundance can be visualized by the dot color/size. An alternate way for visualization of complex mass spectrometric data is the use of DBE versus carbon number (C#) diagrams, which are related but not equivalent to VK diagrams. They are often interchangeably used but provide slightly different information; while a DBE vs C# plot directly reflects the molecular size and the degree of condensation, it does not provide heteroatom (oxygen) content unless the plots are individually drawn for different heteroatomic classes. In contrast, this information is obtained by VK diagrams, but the molecular size is not directly obtained because the plot is a projection of the atomic H/C and O/C ratios, and thus, multiples of the same elemental formulas are projected to the same coordinates.

**Figure 5:**
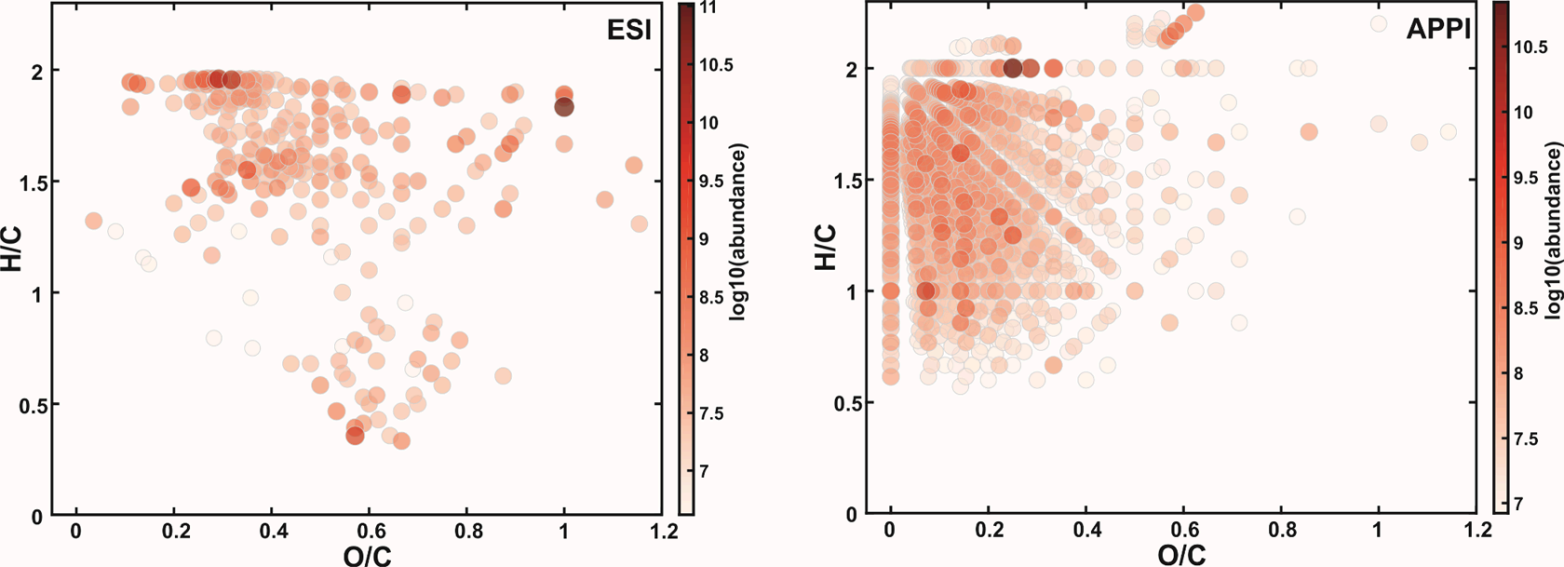
VK diagrams (color coded for relative abundance, logarithmic scale) of the oxygen-class compounds detected in baijiu sample B1 with 12 T FT-ICR MS by using negative-ion ESI or positive-ion APPI.

**Figure 6:**
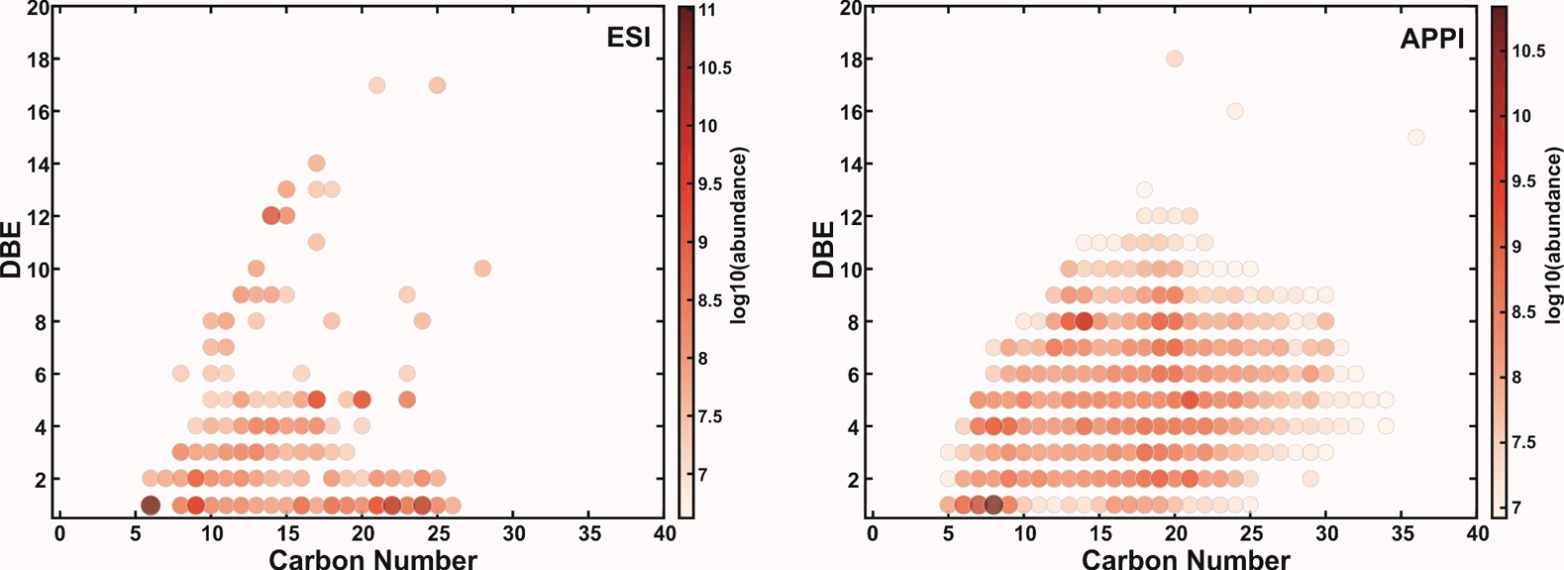
DBE vs carbon number plots (color coded for relative abundance, logarithmic scale) of the oxygen-class compounds detected in baijiu sample B1 with 12 T FT-ICR MS using negative-ion ESI or positive-ion APPI.

In this work, both VK and DBE vs C# diagrams were used for overall chemical composition visualizations of the baijiu liquor samples, obtained either by (−)ESI or (+)APPI (Figures 5 and 6 for the B1 sample; Figures S4 and S7 for the other samples). In the VK diagrams, several species at the top left corner correspond to aliphatic compounds, such as fatty acids, esters, and/or alcohols, having a low oxygen content (Figure 5 and Figures S4 and S5). The species at the top right corner with high H/C and O/C ratios correspond to carbohydrates (polyols). The species located in the middle of the diagram (H/C ≈ 0.5–1; O/C ≈ 0.4–0.8) represent phenolic structures. Both ESI and APPI ionize aliphatic compounds (ESI mainly acids and APPI mainly esters), but only APPI ionizes hydrocarbons (O/C = 0) and other condensed aromatic compounds. Therefore, ESI and APPI are good complementary techniques to study baijiu samples. The same can be seen from the DBE versus C# plots (Figure 6 and Figures S6 and S7). No major differences were obtained between different baijiu samples, by either VK or DBE vs C# diagrams. The only exception was sample B1, which had a much higher content of condensed aliphatic and aromatic (phenolic) compounds than the other liquors (see Figures S4 and S6). Liquor B1 is a light aroma baijiu, which is typically characterized by floral and sweet notes and a fresher palate as compared to strong flavor baijiu liquors.

## 4 Conclusions

Baijiu is a traditional distilled Chinese spirit produced by the fermentation of sorghum, wheat, or other grains. It is chemically a complex organic mixture, containing hundreds or even thousands of compounds, depending on the origin and production methods applied. Here, DI FT-ICR mass spectrometry, coupled with negative-ion ESI and positive-ion APPI, was employed for comprehensive chemical fingerprinting of 10 commercial baijiu liquors. All baijiu samples were dominated by oxygen-containing compounds, especially different acids and esters, which are known to be the most important for distinct favor profiles of different baijiu brands. In addition, several other classes of compounds were detected and identified, including phenolic compounds, some of which were present only in trace amounts and not identified in previous studies. While ESI/APPI FT-ICR MS represents a powerful analytical tool for complex organic mixture analysis, requiring no sample pretreatments, tedious solvent extractions, or chemical derivatizations, the presence of several isomeric species poses a challenge since such compounds cannot be readily differentiated solely based on accurate masses. The use of ion mobility separation and tandem mass spectrometry could overcome some of these limitations. In summary, DI FT-ICR MS can be used for rapid chemical screening of different alcoholic beverages, quality control purposes, new product development, or possible counterfeit product identification.

## Supporting Information Available

Baijiu samples studied in this work, compounds in baijiu liquors identified by positive-ion APPI FT-ICR MS, negative-ion ESI FT-ICR mass spectra of baijiu liquors and the lactic acid standard, positive-ion APPI FT-ICR mass spectra of baijiu liquors, VK diagrams of compounds detected in baijiu liquors with (−)ESI and with (+)APPI, and DBE vs carbon number diagrams of compounds detected in baijiu liquors with (−)ESI and with (+)APPI (PDF)
