# Supplementary material for: High-Resolution Mass Spectrometry-Based Chemical Fingerprinting of Baijiu, a Traditional Chinese Liquor
Source: ACS Omega. 2024 Feb 13;9(8):9443–51. doi: 10.1021/acsomega.3c08993 (PMC10905708; doi:10.1021/acsomega.3c08993)
Supplement: Supplementary file 1 — ao3c08993_si_001.pdf [file ao3c08993_si_001.pdf]

High-Resolution Mass Spectrometry Based Chemical Fingerprinting of Baijiu, a Traditional Chinese Liquor

*Yanning Dou, Marko Mäkinen & Janne Jänis**

Department of Chemistry, University of Eastern Finland, P.O. Box 111, FI-80101 Joensuu, Finland

**Corresponding author e-mail: janne.janis@uef.fi*

Supporting Information

Contents:

Table S1. Baijiu samples studied in this work. 2

Table S2. Identified compounds in baijiu liquors by positive-ion APPI FT-ICR MS. 3

Figure S1. Negative-ion ESI FT-ICR mass spectra of baijiu liquors. 4

Figure S2. Negative-ion ESI FT-ICR mass spectrum of lactic acid standard (1 mM in methanol). 5

Figure S3. Positive-ion APPI FT-ICR mass spectra of baijiu liquors 6

Figure S4. Van Krevelen diagrams for compounds detected in baijiu liquors with (–) ESI. 7

Figure S5. Van Krevelen diagrams for compounds detected in baijiu liquors with (+) APPI. 8

Figure S6. DBE vs carbon number diagrams for compounds detected in baijiu liquors with (–) ESI. 9

Figure S7. DBE vs carbon number diagrams for compounds detected in baijiu liquors with (+) APPI....................10

Table S1. Baijiu samples studied in this work.

Code	Brand name	Class	Alcohol content (% ABV)	Notable ingredients
**B1**	Fenyangwang	Light aroma	42	Sorghum, wheat
**B2**	Xiangquan	Mixed aroma	42	Sorghum, wheat, rice,
**B3**	Taishan	Strong aroma	50	Sorghum, wheat
**B4**	Bainiandianpu	Strong aroma	50	Sorghum, wheat
**B5**	Jinluzhou	Strong aroma	52	Sorghum, wheat
**B6**	Niulanshan aging	Strong aroma	42	Sorghum
**B7**	Niulanshan	Strong aroma	52	Sorghum, wheat
**B8**	China Luzhou	Strong aroma	52	Sorghum, wheat
**B9**	Sanjiaoye Qiguogong	Strong aroma	31	Sorghum, wheat
**B10**	Sanjiaoye	Strong aroma	31	Sorghum, wheat

Table S2. Identified compounds in baijiu liquors by positive-ion APPI FT-ICR MS.

Baijiu sample					1	2	3	4	5	6	7	8	9	10
m/z	Ion formula[1]	Compound(s)	DBE	Error[2] (ppm)	Relative abundance (%)									
104.062052	C_8H_8	Styrene	5	0.051	0.0000	0.0000	0.0000	0.0000	0.0000	0.0203	0.0000	0.0000	0.0539	0.0236
108.056966	C_7H_8O	Benzyl alcohol	4	0.059	0.9157	1.9840	4.7368	1.3954	1.2520	3.7302	1.4797	1.0104	7.7262	3.0965
120.093352	C_9H_{12}	Cumene	4	0.050	0.0413	0.1102	0.1770	0.0718	0.0668	0.1764	0.0822	0.0593	0.3285	0.1504
124.051881	$C_7H_8O_2$	Guaiacol	4	0.042	0.1086	0.2094	0.4803	0.1607	0.1415	0.4301	0.2089	0.1177	0.8752	0.3602
136.051881	$C_8H_8O_2$	Methyl benzoate	5	0.063	0.0000	0.0155	0.0000	0.0000	0.0000	0.0000	0.0000	0.0000	0.0327	0.0177
138.067531	$C_8H_{10}O_2$	Tyrosol	4	0.097	0.1420	0.2358	0.0000	0.1720	0.1454	0.4264	0.1562	0.1030	0.8348	0.3753
140.046796	$C_7H_8O_3$	Ethyl furanoate	4	0.103	0.0000	0.0268	0.0000	0.0148	0.0000	0.0380	0.0164	0.0000	0.0789	0.0317
152.083181	$C_9H_{12}O_2$	4-Ethylguaiacol	4	0.089	0.1883	0.0739	0.0000	0.0747	0.0519	0.1054	0.0560	0.0472	0.1965	0.2277
154.062446	$C_8H_{10}O_3$	Hydroxytyrosol/syringol	4	0.088	0.0443	0.0433	0.0000	0.0254	0.0266	0.0581	0.0362	0.0278	0.0842	0.0549
166.098831	$C_{10}H_{14}O_2$	Wine lactone	4	0.103	0.0230	0.0165	0.0000	0.0000	0.0128	0.0213	0.0172	0.0159	0.0487	0.0287
182.072616	$C_{13}H_{10}O$	Xanthene	9	0.079	0.0198	0.0324	0.0000	0.0143	0.0162	0.0409	0.0355	0.0156	0.0522	0.0303
184.088266	$C_{13}H_{12}O$	Diphenylmethanol	8	0.049	0.0841	0.1256	0.1921	0.0900	0.0698	0.1873	0.1286	0.0606	0.3094	0.1359
212.083181	$C_{14}H_{12}O_2$	Dihydroxystilbene	9	0.045	0.0256	0.0200	0.0000	0.0000	0.0000	0.0303	0.0000	0.0000	0.0625	0.0242
214.098831	$C_{14}H_{14}O_2$	Naphthyl butyrate	8	0.040	0.2588	0.2332	0.0000	0.1550	0.1533	0.3715	0.4284	0.1690	0.7790	0.4897
220.145781	$C_{14}H_{20}O_2$	Benzyl heptanoate	5	0.049	0.0477	0.0526	0.0000	0.0235	0.0299	0.0705	0.0633	0.0224	0.1216	0.0695
230.202902	$C_{17}H_{26}$	Gonane	5	0.051	0.0341	0.0371	0.0000	0.0194	0.0139	0.0479	0.0318	0.0206	0.0704	0.0456
244.218552	$C_{18}H_{28}$	Nonylindane	5	0.032	0.0437	0.0452	0.0000	0.0000	0.0000	0.0508	0.0541	0.0432	0.0781	0.0462
256.218552	$C_{19}H_{28}$	Unidentified	6	0.049	0.0362	0.0438	0.0000	0.0000	0.0000	0.0382	0.0406	0.0213	0.0778	0.0395
262.229117	$C_{18}H_{30}O$	Octadecatrienal	4	0.022	0.0427	0.0760	0.0000	0.0310	0.0259	0.0760	0.3341	0.2317	0.0757	0.0443
264.244767	$C_{18}H_{32}O$	Linolenyl alcohol	3	0.028	0.1005	0.2298	0.0000	0.0622	0.0555	0.1917	0.3033	0.2179	0.2412	0.0167
272.249852	$C_{20}H_{32}$	Stemarene(diterpene)	5	0.023	0.2239	0.1903	0.1859	0.0954	0.0613	0.1360	0.2666	0.0736	0.2153	0.1620
278.224032	$C_{18}H_{30}O_2$	Linolenic acid	4	0.027	0.0587	0.0810	0.0000	0.0245	0.0177	0.0915	0.1196	0.0298	0.0876	0.0461
284.249852	$C_{21}H_{32}$	Diterpene (unidentified)	6	0.027	0.0452	0.0561	0.0000	0.0372	0.0171	0.0660	0.1187	0.0362	0.0970	0.0585
286.265503	$C_{21}H_{34}$	Diterpene (unidentified)	5	0.014	0.0833	0.1407	0.1826	0.0945	0.0363	0.1466	0.3233	0.0909	0.2326	0.1946
294.255332	$C_{19}H_{34}O_2$	Methyl linoleate	3	0.032	0.0482	0.0580	0.0000	0.0000	0.0281	0.0649	0.1104	0.0195	0.0769	0.0455
306.255332	$C_{20}H_{34}O_2$	Incensole	4	0.026	0.0897	0.3950	0.0000	0.0345	0.0297	0.2087	0.2929	0.1853	0.1306	0.0455
308.270982	$C_{20}H_{36}O_2$	Ethyl linoleate	3	0.048	0.0420	0.1634	0.0000	0.0356	0.0336	0.0456	0.7809	0.4848	0.0564	0.0146
316.203296	$C_{20}H_{28}O_3$	Cafestol	7	0.017	0.5022	0.0000	0.0000	0.0000	0.0000	0.0000	0.0000	0.0907	0.0000	0.0000
318.291717	$C_{22}H_{38}O$	4-Hexadecylphenol	4	0.052	0.0285	0.0276	0.0000	0.0182	0.0000	0.0702	0.0279	0.0175	0.0501	0.0248
328.312453	$C_{24}H_{40}$	Unidentified	5	0.012	0.0994	0.1717	0.2287	0.1361	0.0882	0.2537	0.1905	0.0950	0.3737	0.2069
336.208382	$C_{23}H_{28}O_2$	Phenolic (unidentified)	10	0.028	0.0000	0.0000	0.0000	0.0224	0.0182	0.2586	0.0255	0.2156	0.8577	0.2690

[1] For radical cations.

[2] Average error for ten baijiu samples.

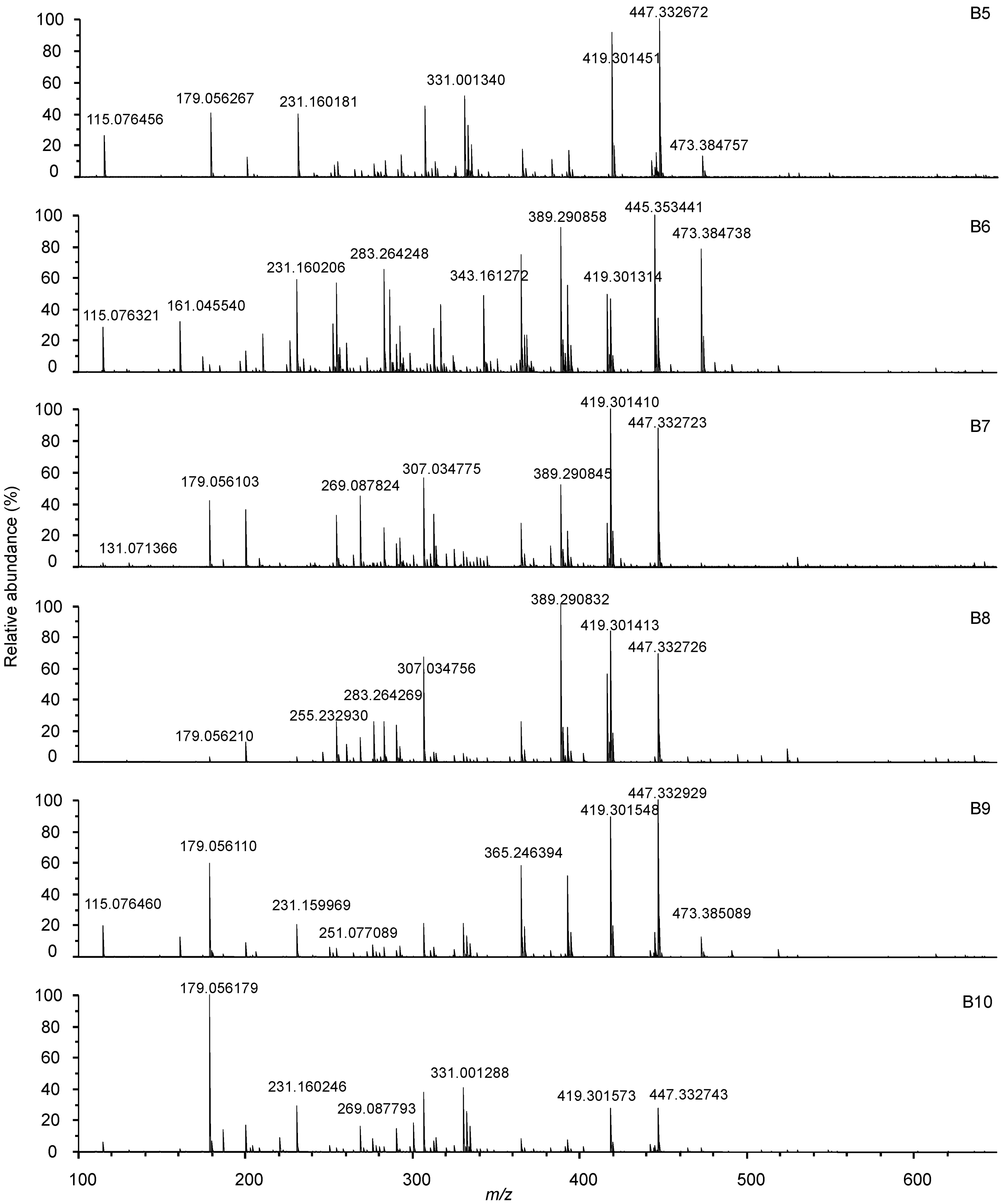

Figure S1. Negative-ion ESI FT-ICR mass spectra of baijiu liquors.

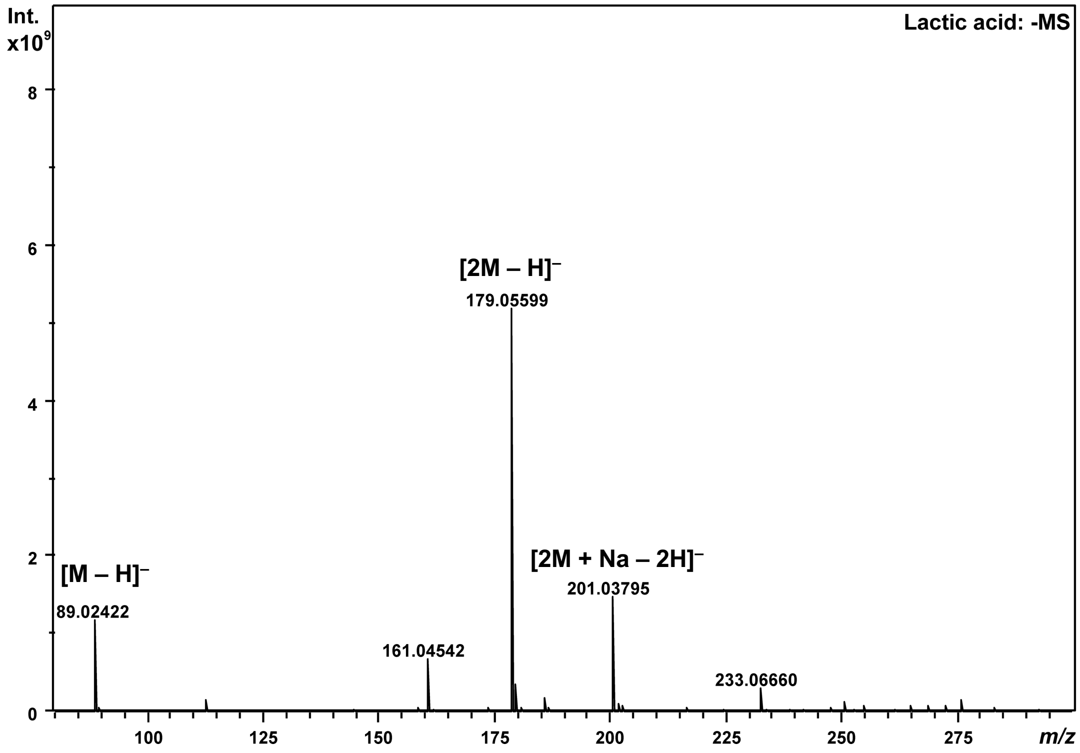

Figure S2. Negative-ion ESI FT-ICR mass spectrum of lactic acid standard (1 mM in methanol).

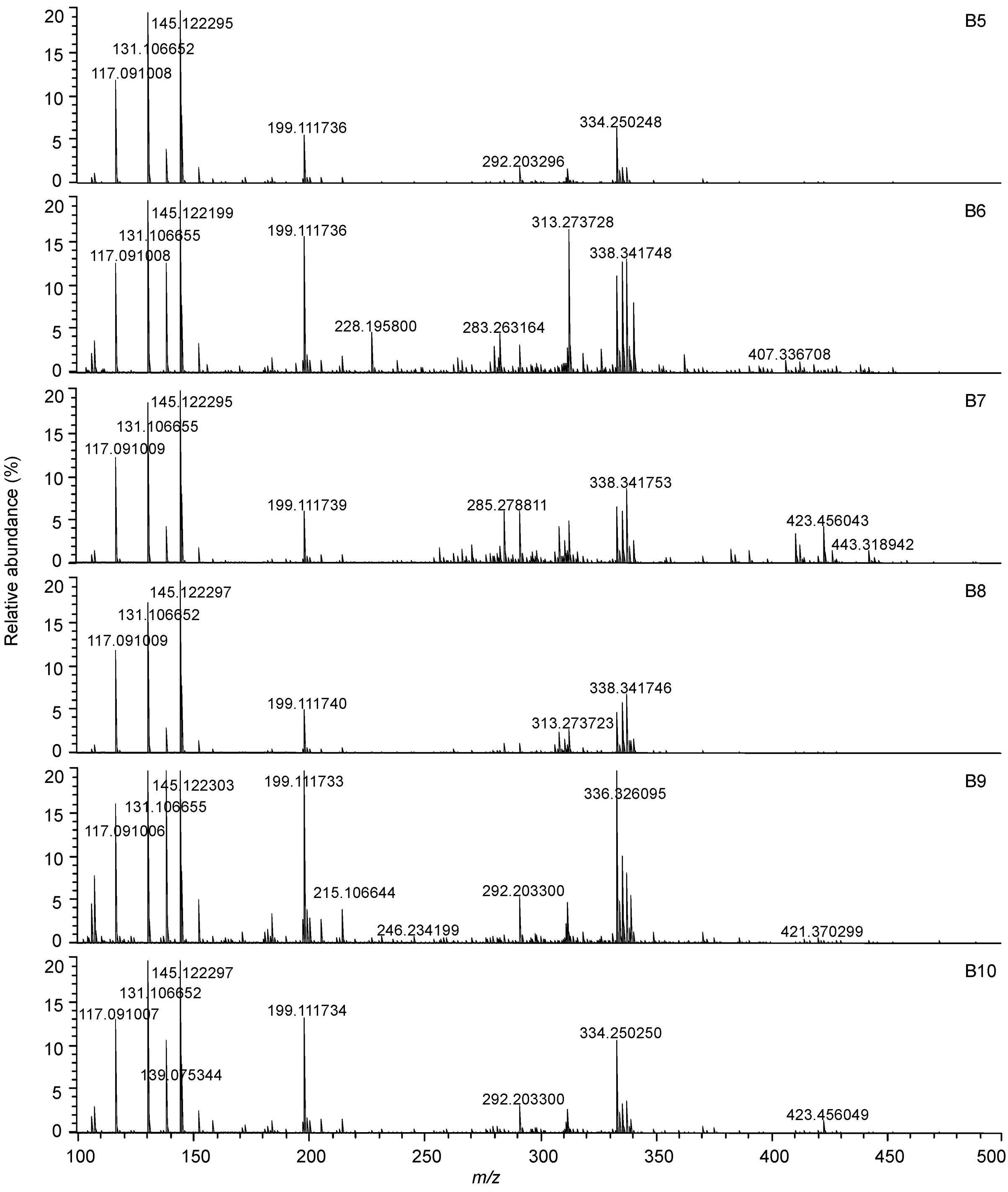

Figure S3. Positive-ion APPI FT-ICR mass spectra of baijiu liquors.

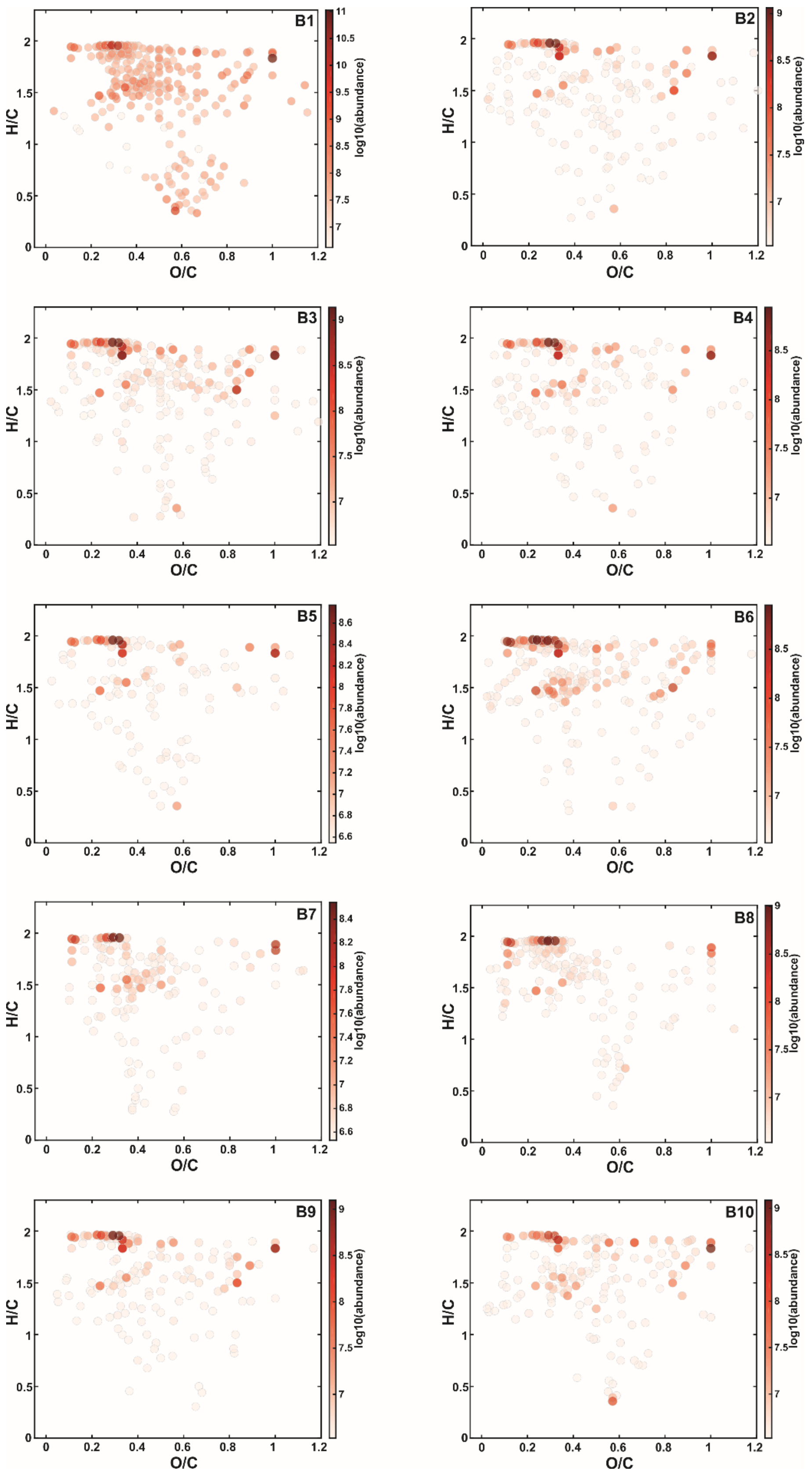

Figure S4. Van Krevelen diagrams for compounds detected in baijiu liquors with (–) ESI.

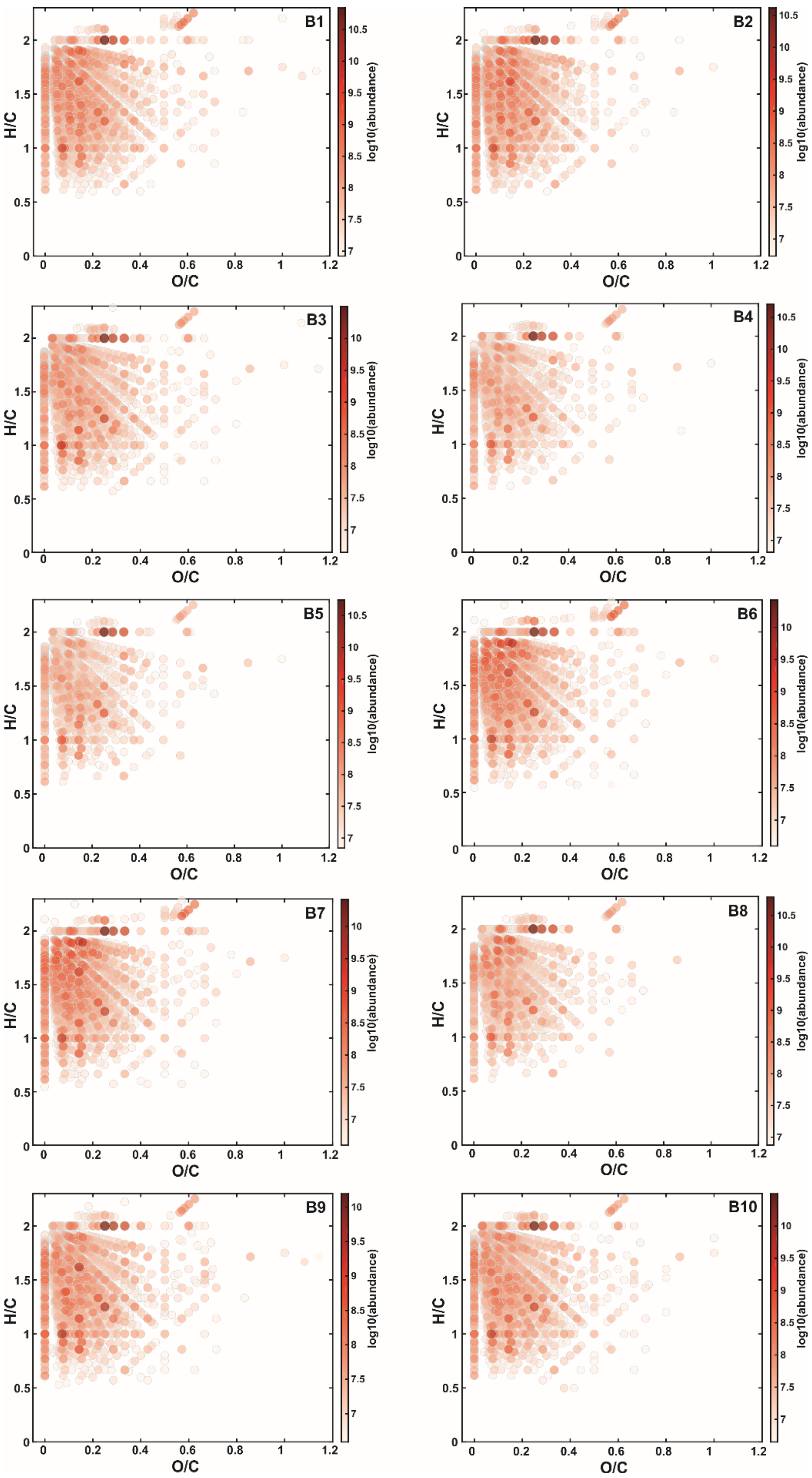

Figure S5. Van Krevelen diagrams for compounds detected in baijiu liquors with (+) APPI.

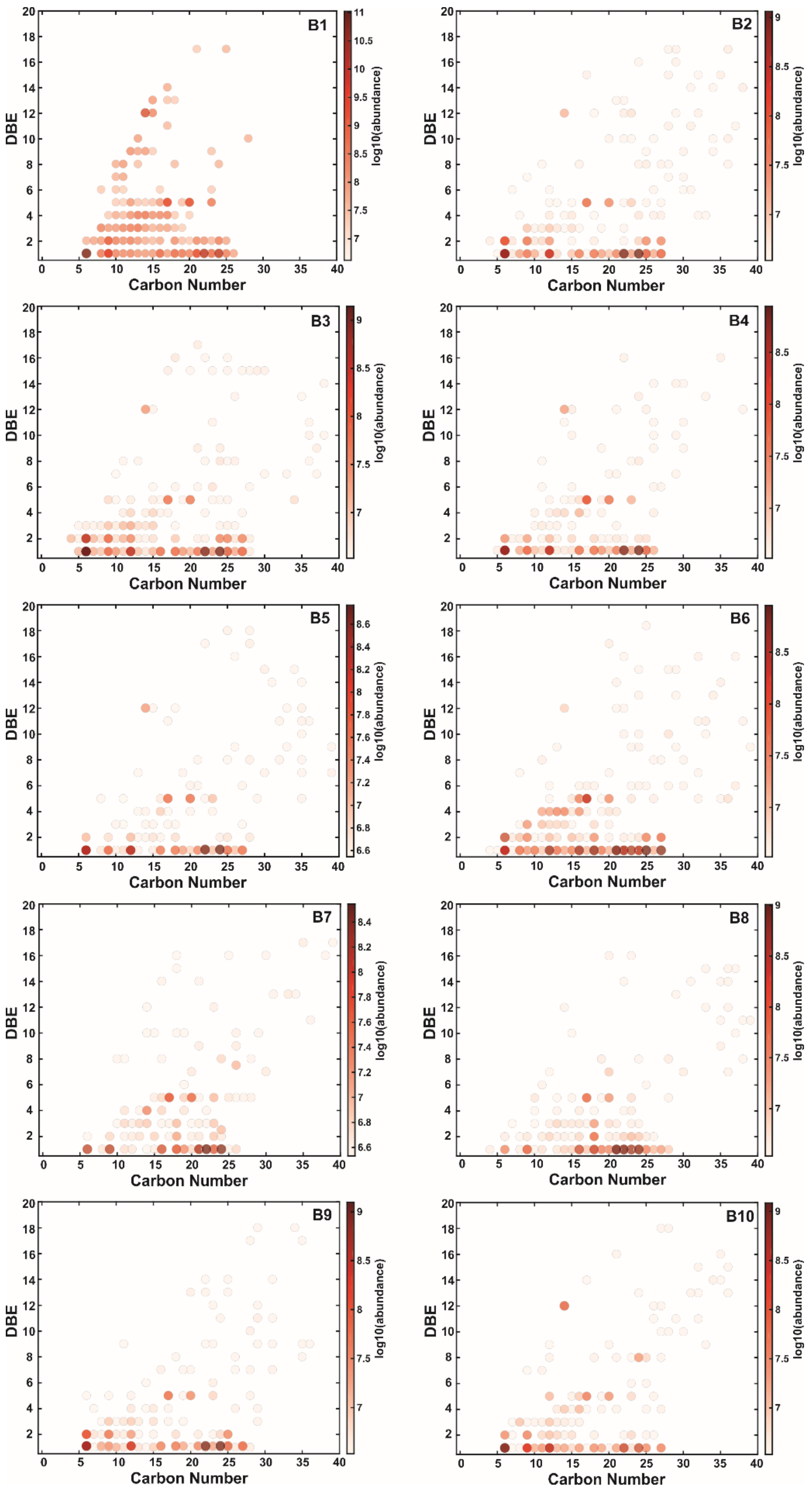

Figure S6. DBE vs C# diagrams for compounds detected in baijiu liquors with (–) ESI.

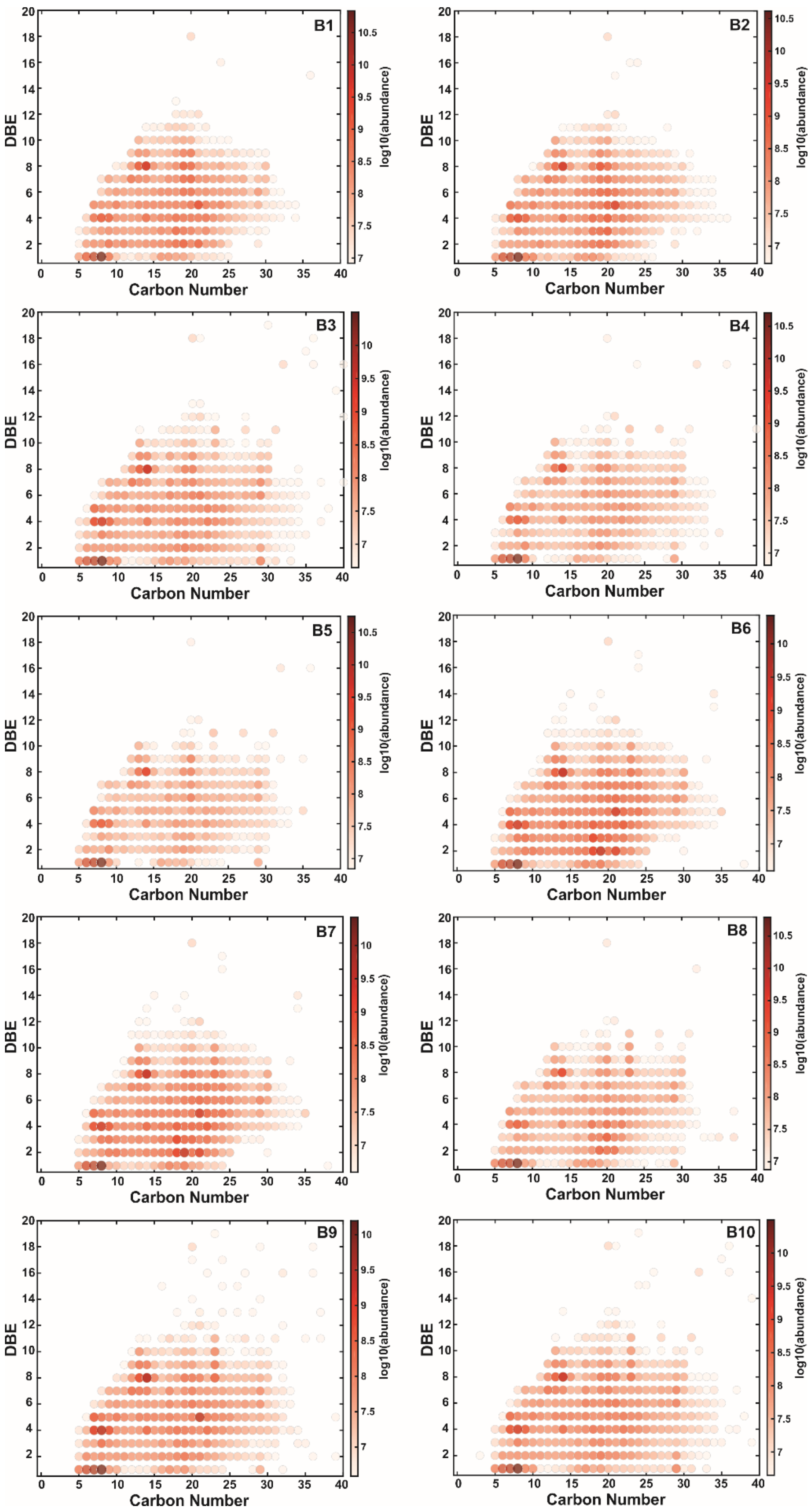

Figure S7. DBE vs C# diagrams for compounds detected in baijiu liquors with (+) APPI
